# Trends in dengue research in the Philippines: A systematic review

**DOI:** 10.1371/journal.pntd.0007280

**Published:** 2019-04-25

**Authors:** Kristal An Agrupis, Michelle Ylade, Josephine Aldaba, Anna Lena Lopez, Jacqueline Deen

**Affiliations:** Institute of Child Health and Human Development, National Institutes of Health, University of the Philippines, Manila, Philippines; Institute for Disease Modeling, UNITED STATES

## Abstract

Dengue is an important public health problem in the Philippines. We sought to describe the trends in dengue research in the country. We searched four databases and identified published studies on dengue research in the Philippines during the past 60 years. We reviewed 135 eligible studies, of which 33% were descriptive epidemiologic studies or case series, 16% were entomologic or vector control studies, 12% were studies on dengue virology and serologic response, 10% were socio-behavioral and economics studies, 8% were clinical trials, 7% were on burden of disease, 7% were investigations on markers of disease severity, 5% were on dengue diagnostics, and 2% were modeling studies. During the last decade, dengue research in the Philippines has increased and evolved from simple descriptive studies to those with more complex and diverse designs. We identified several key topics where more research would be useful.

## Author summary

Dengue is a disease caused by four separate but related viruses transmitted by mosquitos. In this systematic review, we aimed to describe dengue research in the Philippines, where the disease is of great concern, to better understand the types of dengue research and the main findings and important gaps. We identified 135 studies that described dengue research in the Philippines during the past 60 years. Our review showed that in the early years, dengue studies were mainly simple descriptive studies and case reports. Recently the types of investigations have become more complex and diverse, reflecting advancement in local research capacity and infrastructure but more research activity would be beneficial in several areas.

## Introduction

Dengue is a mosquito-borne, acute febrile illness that is an important public health problem in tropical countries. In the early 1950’s, the disease was described in the Philippines as hemorrhagic fever or infectious acute thrombocytopenic purpura [1, 2]. Dengue continues to cause considerable concern in the country because of its widespread endemicity, the minimal success of vector control strategies, the possibility of severe disease caused by sequential infection by a different serotype, the potential for fatal outcomes and the consequent social and economic burden. The four dengue virus serotypes circulate in the country where the disease is predominantly reported among children [3].

Findings from dengue studies could provide policy-makers with information needed for rational decision-making regarding dengue preventive and control efforts. The focus of dengue research may vary widely. This could include basic laboratory research, the estimation of dengue seroprevalence and incidence; the assessment of risk factors for severe disease; the quantification of its economic burden; the elucidation of local transmission and epidemiology; the development of improved diagnostic tests or the evaluation of interventions.

We reviewed published studies on dengue research in the Philippines during the past 60 years. The objective of the review is to better understand the trends in dengue research and the findings from these studies. The results of the review could provide an impression of local capacity and infrastructure for dengue research and help determine important knowledge gaps. These gaps need to be identified since research interest and support for funding can only be achieved if scientists, decision makers and other stakeholders are able to understand developments related to the disease and recognize areas where more information is needed.

## Methods

The Philippines is an archipelago of 7,107 islands and is located in the western Pacific Ocean in Southeastern Asia. The population of the Philippines in 2015 was 100,981,437 [4]. Philippine health status indicators show that the country lags behind most of Southeast and North Asia in terms of health outcomes [5]. Communicable diseases continue to be major causes of morbidity and mortality in the country. Health care in the Philippines is provided through a mixed public-private system.

This systematic review was conducted according to the Preferred Reporting Items for Systematic Review and Meta-Analyses (PRISMA) guidelines [6]. In June 2018, we searched articles on PubMed, the Cochrane Library, ScienceDirect and the Health Research and Development Information Network (HERDIN) from 1 January 1958 to 31 December 2017 combining MeSH and free-text terms for the following: dengue, “dengue fever”, “hemorrhagic fever”, “dengue hemorrhagic fever”, “dengue shock syndrome”, DF, DHF, DSS and Philippines without any language or age restrictions. The search on HERDIN, an electronic database of health research in the Philippines, was done to ensure that articles from local journals not indexed on international databases are included. The completed PRISMA checklist (S1 Table) is shown in the Supporting information. There is no protocol for this systematic review.

The articles were compiled in Endnote (Thomson Reuters, San Francisco, CA, USA). Titles and abstracts were screened for eligibility. Published articles on dengue research in the Philippines and on Filipinos that reported objectives, methods and results or descriptive epidemiologic and case reports were included.

We excluded unpublished articles, studies that were not focused on dengue or not focused on the Philippines, those reporting aggregated results from various countries or analysis of a global or regional collection of viral isolates and specimens from which findings specific to the Philippines could not be retrieved, those reporting the same data from another publication (duplicates), reviews and updates (not original research), meeting or news reports, program descriptions, commentaries, guidelines on dengue (prevention, treatment or diagnosis) and studies on expatriates and non-Filipinos. Towards the goal of assessing the broad picture of dengue research in the Philippines, we included studies that met the basic standard requirements and did not exclude studies based on methodology or risk of bias or selective reporting.

The relevant full papers were downloaded and reviewed in detail. Information from each eligible paper was extracted and entered into an Excel spread sheet (Microsoft Office 2007, Seattle, WA, USA). These included the study title, the year of publication, the journal, the study site primary location, type of study, brief methods and study findings. The summary measures were descriptive.

We compared the annual number of Philippine-related dengue publications with other markers. As a measure of economic growth in the country, we assessed the Philippine Gross Domestic Product (GDP) per capita (in current US dollars) in 1960 (the earliest year data was available) and in 2017 [7]. For comparison, we also obtained the annual number of publications worldwide on PubMed combining the terms: dengue, “dengue fever”, “hemorrhagic fever”, “dengue hemorrhagic fever”, “dengue shock syndrome”, DF, DHF, DSS, from 1958 to 2017, without location, language or age restrictions.

## Results

**Fig 1:**
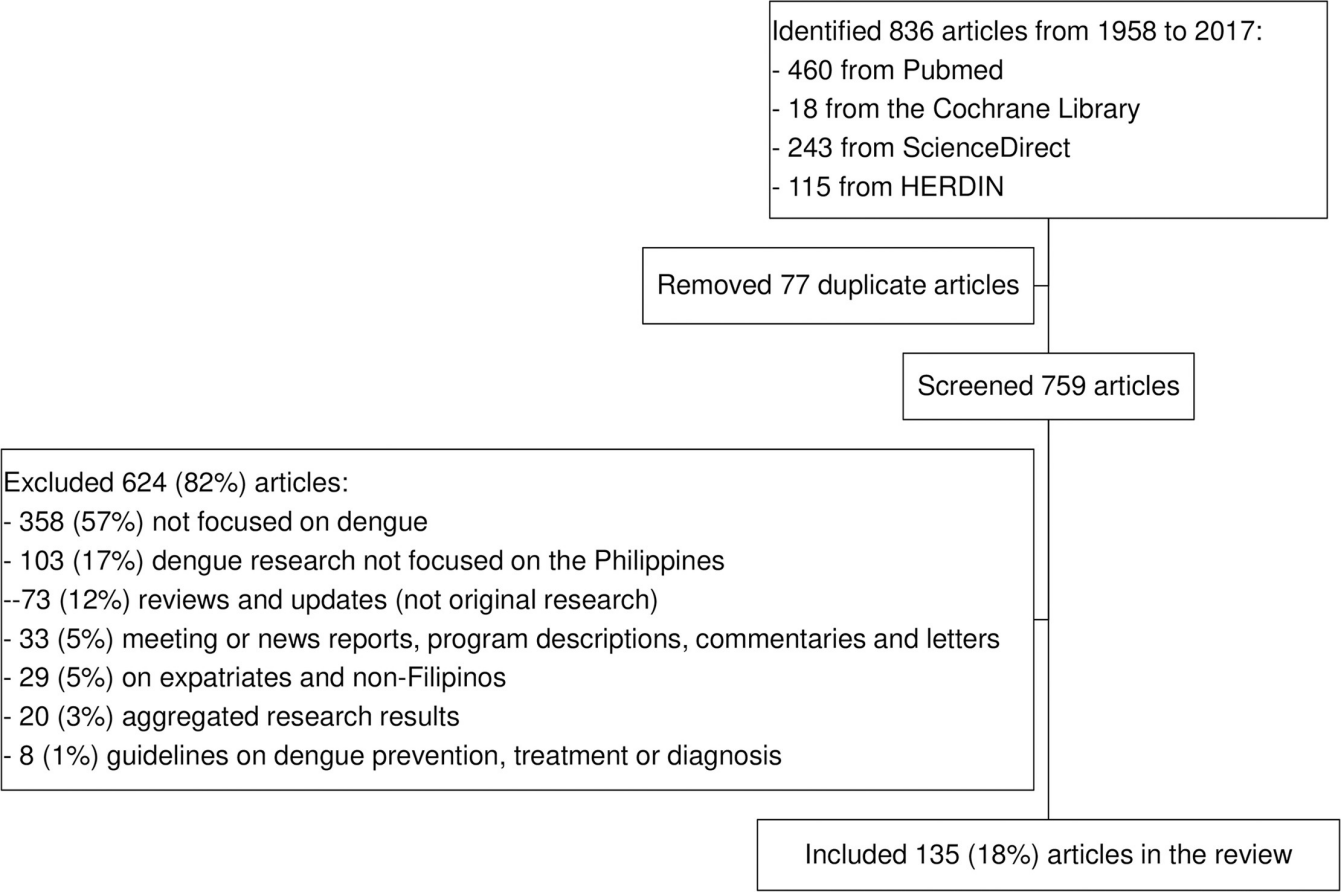
Selection of articles included in the analysis.

We identified 836 published articles on dengue research in the Philippines during the past six decades (Fig 1). We removed 77 duplicates and screened the titles and abstracts of 759 articles, of which 624 (82%) were excluded and 135 (18%) full text articles were downloaded and reviewed. The 135 articles were classified as follows: 44 (33%) descriptive epidemiologic studies or case series [8–51], 21 (16%) entomologic or vector control studies [52–72], 16 (12%) studies on dengue virology and serologic response [73–88], 13 (10%) socio-behavioral and economics studies [89–101], 11 (8%) clinical trials [102–112], 10 (7%) on burden of disease [113–122], 10 (7%) investigations on markers of disease severity [123–132], 7 (5%) on dengue diagnostics [133–139], and 3 (2%) modeling studies [140–142]. The majority (102/135, 76%) of the dengue research locations were in Metro Manila.

**Fig 2:**
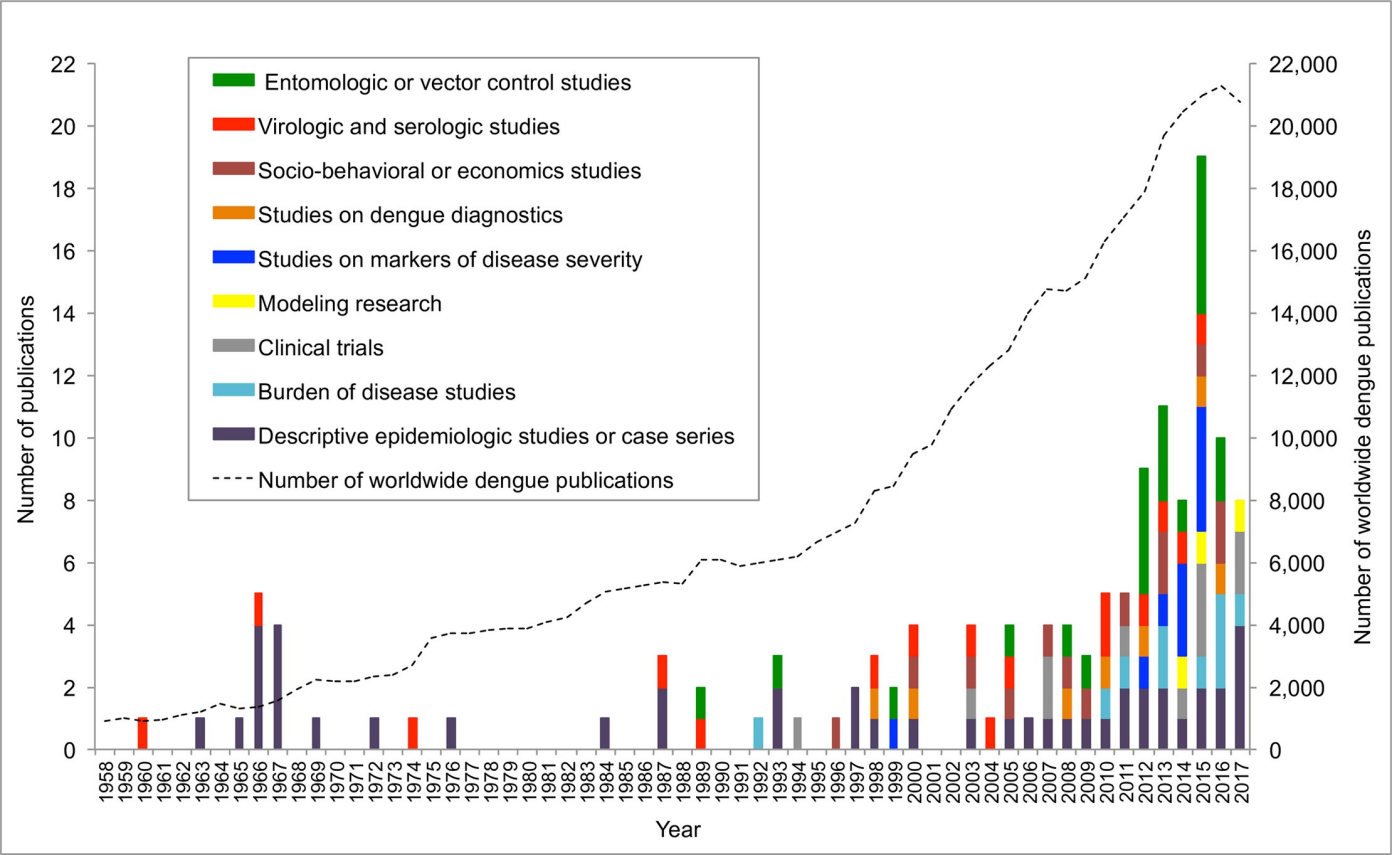
Comparison of annual number of Philippine and worldwide dengue publications.

We assessed the annual number of Philippine dengue studies, by study type and year of publication, and compared this with the annual number of dengue publications worldwide (Fig 2). There were very few articles on dengue research in the Philippines published during the early decades but an increasing annual number in recent years, peaking at 19 articles in 2016. This was associated with an increase in the Philippine GDP per capita from $254 in 1960 to $2,989 in 2017. In comparison, there was a dramatic rise in the annual number of worldwide dengue publications from around 900 articles in 1958 to over 20,000 in 2017 (Fig 2).

### Descriptive epidemiologic studies and case series

The most common studies during the 1960’s were descriptive and these types of studies continue to be published in recent years. The 44 publications included in this category described demographic, clinical and laboratory findings in Filipino patients with suspected or confirmed dengue in hospital or community settings [8–51]. One study of 100 patients who died of clinically-diagnosed dengue hemorrhagic fever reported necropsy findings of intravascular thrombosis and hemorrhages; dengue virus (DENV) was isolated in 32 per cent of the patients [18]. A re-analysis of dengue experimental infection studies in the 1920’s allowed the calculation of an average incubation period for dengue infection of about 6 days [33]. One article described the dengue prevention and response strategies applied after a natural disaster, Typhoon Haiyan that occurred in 2013 [44] while another paper characterized hospital admissions to a tertiary care hospital, including dengue cases, after the typhoon [47]. Five studies assessed the correlation between dengue fever and climate or weather patterns [34, 35, 40, 41, 51]. Longer-term comparative reporting and analysis of dengue fever from around the country would be useful to assess geographic and temporal epidemiologic patterns, risk factors for severe disease, variations in clinical management and changes in case-fatality rates.

### Entomologic and vector control studies

These studies help improve our understanding of the dengue vectors, which could be useful in developing effective control strategies. Of the 21 articles in this category [52–72], six investigated dengue mosquito vector key breeding sites and potential interventions [52, 56–58, 60, 64], three described the response to or efficiency of vector control measures introduced in communities [54, 59, 61], five assessed the larvicidal activity of various agents against *Aedes aegypti* [55, 62, 65, 68, 70], three explored the characteristics and behavior of *Ae*. *aegypti* or *Ae*. *albopictus* [63, 67, 72], one quantified vertical transmission of dengue viruses in *Ae*. *aegypti* [66], two described the population and genetic changes of *Ae*. *aegypti* populations during the dry and wet seasons [53, 69] and one investigated the role of different water-holding containers on the development of *Ae*. *aegypti* [71]. As newer strategies become available (e.g. mosquito sterilization and *Wolbachia*-based approaches), it will be important to investigate these vector control methods in the country.

### Studies on dengue virology and serologic response

In 1960, an article described how viruses isolated from specimens collected in Manila (12 from human sera and 2 from wild-caught mosquitoes) were adapted to suckling mice and shown to be dengue viruses [73]. This was followed by the publication of 15 studies on virologic and serologic aspects of dengue in the Philippines [74–88]. These included one from 1974 reporting how antibody assessments of sera collected from nine participants of dengue experimental infection studies in the 1920’s showed that DENV 1 and 4 were transmitted in these experiments [75]. Several studies described the isolation of various dengue serotypes circulating in the community [76, 77, 79, 81, 84]. A paper compared the nucleotide and amino acid sequences of the nonstructural-1 gene of dengue virus serotype 3 isolated in Metro Manila [78] and another described the molecular epidemiology of DENV 2 [82]. Two studies assessed the presence of dengue antibodies among monkeys in the Philippines suggesting possible sylvatic transmission cycles [80, 86]. In another study, flow cytometric analysis of peripheral blood samples from clinically suspected dengue cases found that B cells are a major replication site for dengue viruses [83]. More recent studies described the continued circulation of a single genotype of DENV 2 in the Philippines [87] and the modulatory effects of compounds on dengue virus infected cells [88]. Continued monitoring of the circulating dengue viruses in the Philippines would help in understanding better the epidemiology of the disease.

### Socio-behavioral and economics studies

Together with epidemiologic studies that quantify the incidence and seroprevalence of disease, socio-behavioral and economic research provides information on how dengue impacts affected communities. There were nine dengue socio-behavioral studies [89–93, 95, 96, 98, 100]. Six assessed dengue-related knowledge and preventive practices in different communities [89, 90, 92, 93, 96, 98]. Two were multi-country studies that included the Philippines and used questionnaires and focus group discussions to assess policymakers’ views on dengue and the need for a dengue vaccine [91] and health care providers’ use of dengue clinical guidelines [95]. One documented anecdotal use of a local herb in the treatment of dengue [100]. In light of the recent dengue vaccination controversy in the country, a study on policymakers’ understanding of dengue's complicated pathophysiology and immunologic responses would be useful in addressing unresolved issues and also for considering what would be needed when implementing future dengue control strategies.

There were four economics studies [94, 97, 99, 101]. One published in 2008, prior to the licensure of the first dengue vaccine, used a contingent valuation survey and found a high willingness to pay and household demand for a dengue vaccine [94]. In another study, investigators assessed the economic and disease burden of dengue in 12 Southeast Asian countries [97]. For the Philippines, they calculated the direct cost for each hospitalized and ambulatory dengue case (in 2010 US dollars) of $177 and $47, respectively, plus indirect costs of $36 and $17, respectively. In a later publication, an annual average of 842,867 clinically diagnosed dengue cases in the Philippines was estimated, with direct medical costs (in 2012 US dollars) of $345 million ($3.26 per capita) [99]. The potential cost-effectiveness of a dengue vaccination program was discussed in another paper [101]. It will be useful to estimate the economic benefits of new dengue control methods in the country, as they become available.

### Clinical trials

Of the 11 publications on dengue-related clinical trials, four were on therapeutic interventions [102–105] and seven were on vaccine trials [106–112]. The therapeutic interventions assessed included a hemostatic agent [102], fluids [103] and immunoglobulin [104, 105]. Multi-country randomized controlled trials of candidate dengue vaccines included study sites in the Philippines and the seven papers we identified reported on vaccine safety, immunogenicity and efficacy [106–108, 110–112], as well as concomitant dengue and MMR vaccination [109]. As newer dengue vaccines and therapeutics become available, it will be important to investigate these interventions in the country.

### Burden of disease

Ten studies assessed the burden of dengue infections [113–122]. A study from 1992 reported an attack rate of 0.2 dengue cases per 1,000 population for the period of July to December 1990 in Zamboanga city [113]. On a national scale, the annual dengue surveillance data from the Philippines (included among other countries in the World Health Organization Western Pacific Region) showed dengue fever notification rates of 1.5 per 1,000 population in 2010, 1.3 per 1,000 population in 2011 and 1.9 per 1,000 population in 2012 [115, 116, 118]. Another paper quantified epidemiologic trends in dengue disease burden in 5 Asian countries, including the Philippines, over a 30-year period using data from DengueNet and the WHO [122]. The estimated dengue incidence and mortality in the Philippines increased by 24% and 29%, respectively, but the authors acknowledged that implementation of more sensitive surveillance methods over the study period may have contributed to a reporting bias. These data provide an overall picture but are based on routine passive notification, often of clinically diagnosed cases, and may be weakened by incomplete reporting and delays.

Among the burden of disease articles, incidence of laboratory-confirmed symptomatic dengue infections were estimated in several prospective surveillance studies that actively followed a cohort for acute febrile illness [114, 117, 119–121]. Incidence was calculated using the number of new cases arising from the defined cohort as the numerator and the years of observation time contributed by each person in the cohort as the denominator. Table 1 shows the estimated incidence of laboratory-confirmed symptomatic dengue infections from the articles. In the first study, Capeding and co-workers followed 4,441 healthy infants; and dengue infection was confirmed by serotype specific reverse transcriptase-polymerase chain reaction (RT-PCR) in acute-phase sera and dengue IgM/IgG enzyme linked immunosorbent assay (ELISA) in paired acute and convalescent phase sera [114]. The incidence of symptomatic (clinically apparent) infant dengue infections was 16 per 1,000 person-years (Table 1), of which hospitalized episodes occurred at 8 per 1,000 person-years. Serologic testing of serial blood samples from a subset of 250 infants without reported febrile illnesses in 2007 showed an incidence of clinically-inapparent dengue infections (defined as a > 4-fold rise in dengue virus 50% plaque-reduction neutralization titers between two time points with a monotypic pattern), that was 6-fold higher than that of symptomatic infections at 103 per 1,000 person-years (95% CI 64–155). Second, in a multi-center study, 300 healthy children 2 to 14 years at two sites in the Philippines were actively followed for febrile illness and dengue was confirmed using a nonstructural protein 1 (NS1) antigen ELISA in acute serum samples and IgM/IgG ELISA in both acute and convalescent samples [117]. The incidence of confirmed symptomatic dengue infections was 34 per 1,000 person-years (Table 1). In the third study, 854 participants 6 months to over 50 years of age underwent active fever surveillance and annual serological assessment [119]. Acute sera were tested by dengue PCR and acute/convalescent samples by dengue IgM/IgG ELISA to identify symptomatic infections while enrolment and 12-month samples were tested by dengue hemagglutination inhibition assay to identify subclinical infections. The incidence of symptomatic dengue infection was 16 per 1,000 person-years (Table 1) and clinically inapparent dengue infections occurred at 70 per 1,000 person-years (95% CI 54–90). Symptomatic dengue rarely occurred in those older than 15 years. Fourth, two articles reported the incidence of virologically-confirmed dengue in the control group of a multi-center phase 3 trial of a dengue vaccine, including 1,166 participants 2 to 16 years of age at two Philippine study sites [120, 121]. The children were followed for acute febrile illness and dengue infection was confirmed by means of both NS 1 antigen and RT-PCR assays. The incidence of symptomatic dengue infection was 66 per 1,000 person-years (Table 1), of which hospitalized episodes occurred at 7 per 1,000 person-years (95% CI 4–12). In comparison with the national data described above, these incidence data provide a more accurate estimate of the burden of dengue because of the active surveillance in a defined cohort and the laboratory-confirmation of cases. But they are limited by having been conducted at only three sites (Laguna, Metro Manila and Cebu) in the country. The wide differences in incidence of laboratory-confirmed symptomatic dengue infections in the studies (Table 1) are due to the different age groups in the cohort and varying time periods (dengue has seasonal and cyclical epidemic patterns) but may also reflect variations in the dengue force of infection across the sites. Additionally, differences in fever detection methods and diagnostic confirmatory tests may have contributed to the variation in the incidence estimates.

**Table 1:** Estimated incidence of laboratory-confirmed symptomatic dengue infections from studies in the Philippines.

	Location of study	Surveillance period	Study population sample size	Age at enrolment into study	Incidence
Capeding [114]	San Pablo, Laguna	2007 to 2008	4,441	6 to 18 weeks old	16 / 1,000 person-years (95% CI 11–22)
Capeding [117]	San Pablo, Laguna, Metro Manila and Cebu City	2010 to 2011	300	2 to 14 years old	34 / 1,000 person-years (95% CI 15–77)
Alera [119]	Punta Princesa, Cebu City	2012 to 2013	854	6 months to over 50 years old	16 / 1,000 person-years (95% CI 10–26)
L’Azou [120]	San Pablo, Laguna, and Cebu City	2011 to 2013	1,166	2 to 15 years old	66 / 1,000 person-years (95% CI 56–77)

We derived data on dengue seroprevalence in Filipinos from two studies that conducted baseline serologic assessments prior to fever surveillance [119, 120]. First, among participants over 6 months of age in Cebu City, dengue seroprevalence assessed by hemagglutination inhibition assay increased sharply with age [119]. The proportion of participants with a multitypic dengue serologic profile was 40% in the 6 month to 5-year-old age group compared to 99% in the 31 to 50 year olds. Second, baseline dengue seropositivity prior to vaccination, assessed in 604 Filipino children by plaque-reduction seroneutralization assay, was 78% overall and 58%, 75%, 86% and 93% in the 2–4, 5–8, 9–12 and 13–16 year old age group, respectively [120].

### Investigations on markers of disease severity

Ten studies looked for associations between biomarkers and clinical presentation of dengue disease. Eight studies assessed levels of various immune-related or enzymatic biomarkers [123–127, 130–132], while two evaluated the potential role of adiposity [128, 129]. More research is needed to better understand the host characteristics that contribute to dengue disease severity.

### Dengue diagnostics

There are several methods available for the diagnosis of dengue fever, including virus isolation, detection of viral components (RNA or antigen) and serological assays. In the Philippines, RT-PCR is the confirmatory test of choice but RT-PCR is expensive and time consuming, requires technical expertise and high-level laboratory equipment and does not provide immediate results that could be used for patient care. Dengue rapid diagnostic tests are used at the point-of-care but have insufficient sensitivity and specificity. We found seven published studies that assessed various dengue diagnostic tests, including ELISA [133–135, 138], fluorogenic real-time RT-PCR [136] and rapid diagnostic tests [137, 139]. The gold standard used for comparison in these studies was conventional RT-PCR. Definitive diagnosis of dengue is important for the clinical management of patients, disease surveillance and outbreak investigations. A dengue diagnostic assay with sufficient sensitivity and specificity, that is less cumbersome than RT-PCR and with results immediately available for clinical care would be very useful.

### Modeling studies

There were three studies that used modeling techniques to estimate dengue burden and describe disease patterns [140–142]. Using historical epidemiological, environmental, socio-economic and climate data, one study developed prediction models for future dengue incidence in the Philippines [140]. From an analysis of 18 years of dengue surveillance reports in eight countries in Southeast Asia, including the Philippines, investigators found strong patterns of synchronous dengue transmission across the entire region coinciding with elevated temperatures associated with anomalies in Pacific Ocean surface temperatures (Oceanic Niño index) [141]. Another study estimated 794,255 annual dengue episodes and a disease burden of 535 DALYs per million population in the Philippines extrapolated from passive routinely-collected data compared with results from a prospective community-based cohort study at one site [142]. Modeling studies may be useful in the evaluation of dengue interventions or control studies that become available in the future, especially when field studies are not feasible.

## Discussion

We report on published, dengue research in the Philippines during the past 60 years. During the last decade, there have been an increasing number of dengue studies in the Philippines. From the 1960’s to the 1990’s, the studies were mainly descriptive epidemiologic assessments and case series, but during the recent years, the types of investigations have become more complex and diverse. We believe this reflects advancement in local research capacity and infrastructure. The improvement has coincided with an increase in annual GDP per capita. Globally, there has also been an upsurge in dengue-related publications over the recent decades, probably due to an increasing interest in dengue together with its geographic expansion, more research publications from dengue-endemic countries, the assessment of recently developed strategies against the disease, as well as the proliferation of medical journals.

Despite the increase in dengue research in the Philippines, we identified several dengue knowledge gaps. The vast majority were descriptive short-term hospital- or community-based studies. A longer-term comparative assessment of dengue epidemiologic patterns by site and year would be useful to understand the bigger picture of dengue in the country. As newer vector control methods and vaccine and therapeutic interventions become available, it will be important to investigate these strategies in the country. Sociobehavioral, economics and modeling studies related to these future interventions would be important to assess their impact. More studies on basic laboratory research, including continued monitoring of the circulating dengue viruses in the country and dengue serologic response would help to provide a better understanding of dengue epidemiology in the country. The incidence and seroprevalence data are available from a few sites and it is not known whether this is generalizable to other areas of the country.

Aside from these important research areas, it is essential that basic dengue information and updated findings be communicated to policymakers, health workers, academics and other stakeholders. Researchers may need to liaison with the media to avoid miscommunication to the general public. This is especially important to avoid issues arising from misunderstanding when new control measures are implemented. Perhaps the recent controversy that surrounded the dengue vaccination program could have been avoided by prior detailed communication and education for more informed decision-making.

There are several limitations of this review. First, although we searched four databases (including a local repository), it is possible that some publications were missed. Second, there was some overlap in topics covered by some papers and we selected the main theme covered in the classification and assessment of results. Third, although the majority of the articles (117/135 or 87%) included a Filipino author affiliated with a Philippine institution, foreign collaborators led many of the projects for which much of the laboratory work and data analysis were done outside the Philippines. Although dengue research capacity and infrastructure in the Philippines appears to have significantly increased during the recent decades, we are not able to exactly quantify the improvement. As local investigators gain more experience in developing proposals, obtaining grants and implementing research, we hope that more dengue projects will be lead by Filipino scientists. Fourth, this review on identifying dengue research gaps is just one step towards defining specific questions of interest on dengue in the Philippines. There needs to be a fuller engagement of scientists, policymakers and the public and the development of a continuing method to assess the evolving dengue research needs of the country.

The importance of dengue research is justified by the data showing a significant burden of the disease. These studies indicated a symptomatic laboratory-confirmed dengue incidence of 16 to 66 per 1,000 person-years (depending on the age group, the year when the study was done, the intensity of the surveillance method and the diagnostic method), while the incidence of hospitalized dengue was estimated at 7 to 8 per 1,000 person-years. Furthermore, clinically inapparent or asymptomatic dengue infections occur quite frequently, many folds higher than symptomatic dengue, due to the intense transmission of the virus. The available incidence and seroprevalence data confirm the high endemicity of dengue infections in the country, which results in a heavy socio-economic burden.

The epidemiology of dengue varies in different geographical areas around the world. Describing what is happening in the Philippines can provide a template for other dengue-endemic areas. A standardized protocol could be developed from this and other reviews [143] for those who wish to conduct a similar activity in other dengue-endemic countries. Publishing data on the research needed to improve health care delivery is part of the communication that is central and key to successful implementation of public health programs. This is particularly true in the Philippines where dengue vaccination has recently been in the limelight when it was introduced in 2016 and stopped the year after. Initial introduction and subsequent events that resulted in highly controversial issues were partly due to misunderstanding of dengue's complicated pathophysiology and immunologic responses.

In conclusion, this review showed that dengue studies in the country have increased in number and evolved from simple to more complicated types of investigations. We identified several important areas for increased research efforts. Studies such as this can help raise awareness on the significance of the disease and the need for better treatment and preventive strategies.

## Supporting information

S1 Table PRISMA checklist. (DOCX)
